# Supplementary material for: Asymmetric Synthesis of Quaternary Hydantoins via a Palladium-Catalyzed Aza-Heck Cyclization
Source: J Am Chem Soc. 2025 Nov 14;147(49):44692–8. doi: 10.1021/jacs.5c16022 (PMC12703750; doi:10.1021/jacs.5c16022)
Supplement: Supplementary file 2 [file ja5c16022_si_002.zip › All NMR FID Files/8/8_ProtonNMR/pdata/1/email_TDI01-064 clean xterize_5_1.pdf]

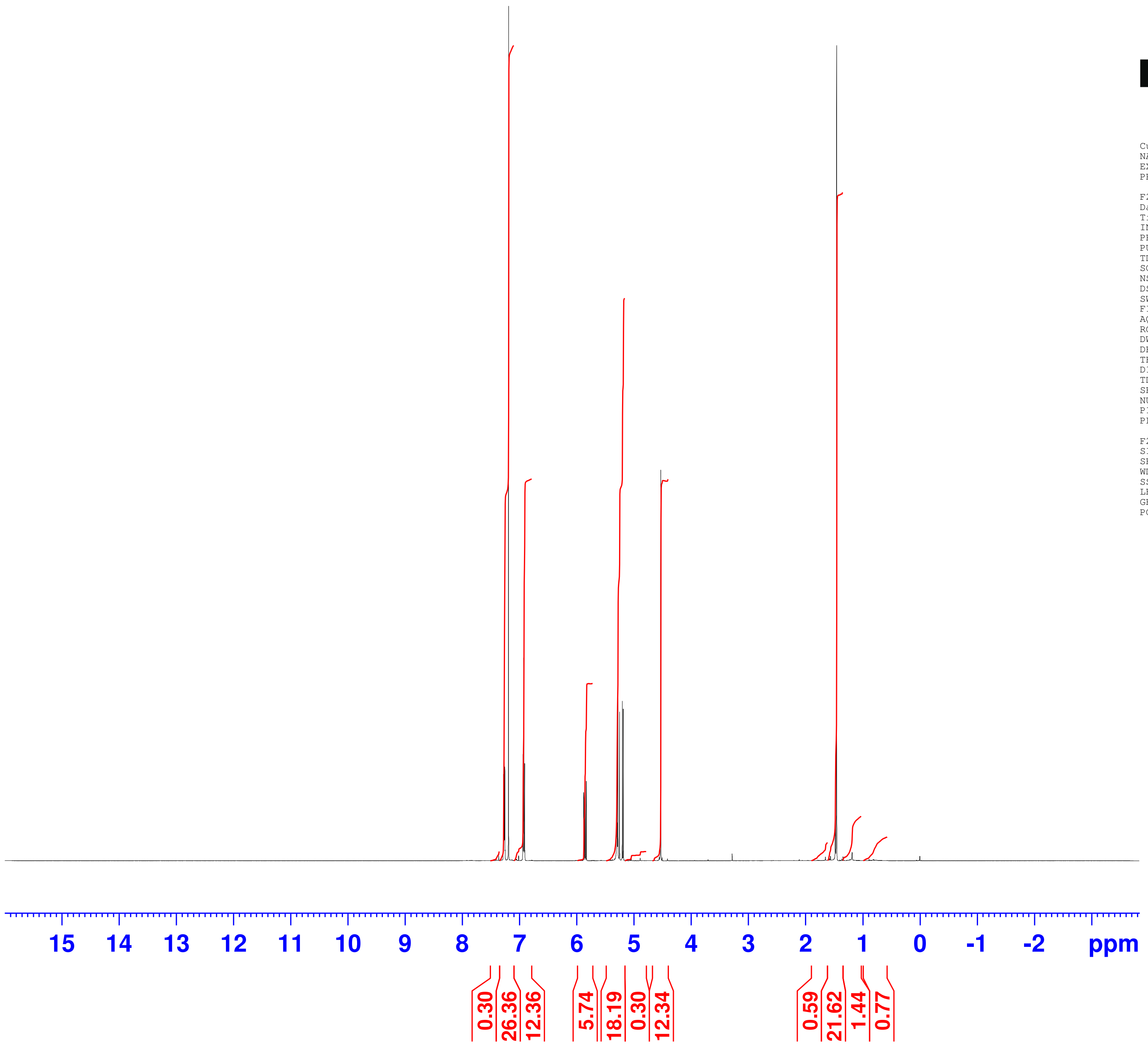


```
Current Data Parameters
NAME     TDI01-064 clean xterize
EXPNO                      5
PROCNO                     1

F2 - Acquisition Parameters
Date_               20250209
Time                   21.11 h
INSTRUM  Avance Neo 600 at University of Delaware
PROBHD   Z185170_0001 (
PULPROG                   zg
TD                     65536
SOLVENT                CDCl3
NS                        16
DS                         2
SWH                11904.762 Hz
FIDRES              0.363304 Hz
AQ                 2.7525120 sec
RG                      12.7
DW                    42.000 usec
DE                     10.61 usec
TE                     298.0 K
D1               10.00000000 sec
TD0                        1
SFO1             600.1337058 MHz
NUC1                      1H
P1                      8.07 usec
PLW1              6.90000010 W

F2 - Processing parameters
SI                     65536
SF               600.1300568 MHz
WDW                       EM
SSB                        0
LB                      0.30 Hz
GB                         0
PC                      1.00
```
